# Exceptional mucocutaneous manifestations with amyloid nephropathy: a case report

**DOI:** 10.1186/s13256-018-1760-6

**Published:** 2018-08-21

**Authors:** Se-Hee Yoon, Jang-Hee Cho, Hee-Yeon Jung, Won-Min Hwang, Sung-Ro Yun, Ji-Young Choi, Sun-Hee Park, Chan-Duck Kim, Mee-Seon Kim, Yong-Lim Kim

**Affiliations:** 1 Division of Nephrology, Department of Internal Medicine, Myunggok Medical Research Institute, College of Medicine, Konyang University, Daejeon, South Korea; 2 Department of Internal Medicine, School of Medicine, Kyungpook National University, Kyungpook National University Hospital, Daegu, South Korea; 3 Department of Pathology, Samsung Changwon Hospital, Sungkyunkwan University School of Medicine, Changwon, South Korea

**Keywords:** Systemic amyloidosis, Sicca syndrome, Nail dystrophy, Renal dysfunction

## Abstract

**Background:**

Amyloidosis is a very rare disease that is difficult to diagnose because of the unspecific early clinical manifestations of the disease. Accurate and early diagnosis is extremely important because the effect of treatment is dependent on the extent of disease progression. Sicca syndrome and nail dystrophy are very rare symptoms of amyloidosis. We report here a case of sicca syndrome and nail dystrophy with renal dysfunction in a 52-year-old Korean woman who was diagnosed as having systemic amyloidosis.

**Case presentation:**

We present the case of a 52-year-old Korean woman complaining of dry mouth and nail dystrophy for 4 months as an initial symptom. A slit lamp examination revealed superficial keratoconjunctival erosion in both eyes. A laboratory test showed anemia, azotemia, and proteinuria. Urine protein electrophoresis showed increased gamma globulin excretion. Serum free light chain of kappa and lambda were increased. Histopathological studies of biopsy specimens of minor salivary glands and kidney revealed deposits of amyloid fibrils. A bone marrow aspiration biopsy showed hypercellular marrow with 5% plasma cells. She was diagnosed as having primary systemic amyloidosis then started on chemotherapy.

**Conclusion:**

Such atypical mucocutaneous manifestations of amyloidosis can serve as important early diagnostic signs with less invasive biopsy confirmation in patients with systemic amyloidosis.

## Background

Amyloidosis is a disease caused by extracellular deposition of insoluble polymeric protein fibrils in tissues and organs. The fibrils have a characteristic β-plated sheet configuration that produces apple-green birefringence under polarized light when stained with Congo red dye [1]. Amyloidosis can develop as either a limited cutaneous or as a systemic disease. Systemic amyloidosis is classified into primary and secondary types. Primary systemic amyloidosis (AL amyloidosis) may be idiopathic or myeloma associated. AL amyloidosis is characterized by the pathological production of fibrillary proteins composed of intact or fragments of monoclonal immunoglobulin light chains which accumulate in tissues [2]. Amyloid proteins can accumulate in virtually every organ with the exception of parenchymal brain tissue. Cutaneous manifestations, such as purpura, petechiae, and ecchymoses, are observed in approximately 50% of cases of AL amyloidosis and provide important diagnostic clues [3]. Amyloidosis is difficult to diagnose because of the unspecific early clinical manifestations. Accurate and early diagnosis is extremely important because the effect of treatment is dependent on the extent of disease progression. Such mucocutaneous manifestations of amyloidosis can serve as important early diagnostic signs with less invasive biopsy confirmation in patients with systemic amyloidosis. Here we report a case of systemic amyloidosis presented with sicca syndrome and nail dystrophy with renal involvement.

## Case presentation

A 52-year-old Korean woman presented to our hospital because of edema and dry mouth. Additional complaints included fatigue, weight loss, and nail dystrophy. She had not had any of these symptoms in her medical history before. She started to complain of dry mouth and nail dystrophy 4 months before admission. During that period she lost 4 kg and felt severe fatigue. Two months before admission, she underwent a health screening in another hospital and received a diagnosis of renal dysfunction and hypothyroidism. There was no fever, rash, Raynaud phenomenon, or articular complaints. She had no medical history, and denied tobacco or alcohol abuse. There was no history of renal disease in her family.

**Fig. 1:**
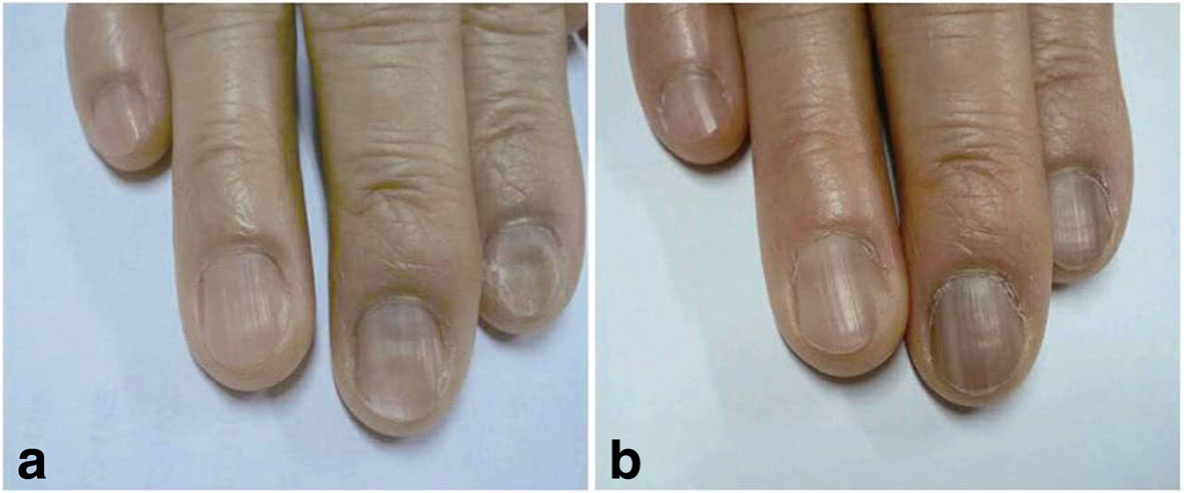
**a** The nails were flattened and revealed onycholysis at admission. **b** The dystrophy of nails improved 8 months later

Her blood pressure was 120/70 mmHg, pulse rate was 62/minute, and her body temperature was 36.8 °C. Her conjunctiva was pale and her oral cavity was dry. Her nails were flattened and revealed onycholysis (Fig. 1a). A KOH test showed no fungi in her nails. Her thyroid gland was not enlarged and superficial lymph nodes were not palpable. There was no hepatosplenomegaly. No abnormality was present in the urological findings and neurologic examinations.

Laboratory findings disclosed the following: hemoglobin 69 g/L, erythrocyte sedimentation rate 53 mm/hour, serum protein 88 g/L, albumin 26 g/L, serum creatinine 298.8 umol/L, and creatinine clearance 15 ml/minute according to Cockcroft and Gault formula. Urine sediment contained 0–2 white blood cells (WBC) and 0–2 red blood cells (RBC) per field. Her 24-hour urinary protein excretion was 0.696 g/day. Antinuclear antibodies, antibodies to SSA and SSB, rheumatoid factor, complement fractions, and cryoglobulins were all negative or within the normal range. In a urine protein electrophoresis, the proportion of urine protein was the following: albumin 87.6%, α1-globulin 4.0%, α2-globulin 1.1%, ß-globulin 0%, and γ-globulin 7.3%. Serum and urine protein immunofixation electrophoresis showed no abnormal finding. Serum free light chain of kappa and lambda was increased (0.307 g/L and 0.192 g/L, respectively). Serum immunoglobulin G (IgG) was raised at 38.55 g/L (normal 7.0–17.0), however, IgA and IgM levels were normal (3.04 g/L and 2.45 g/L, respectively).

**Fig. 2:**
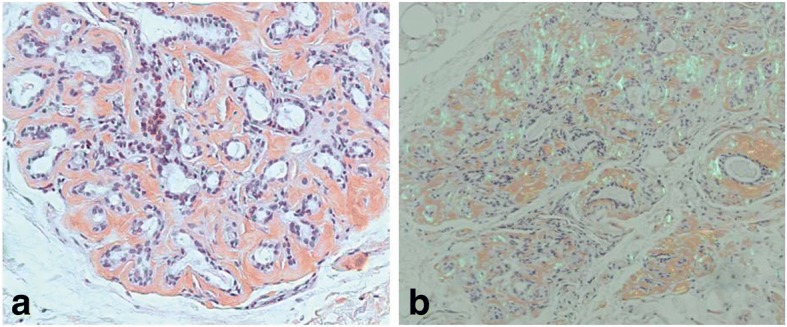
Microscopic feature of lip biopsy showing amyloid deposition around the salivary ducts and atrophy of the salivary duct. **a** Congo red stain, × 100; **b** Congo red stain by birefringence under polarized light, × 100

**Fig. 3:**
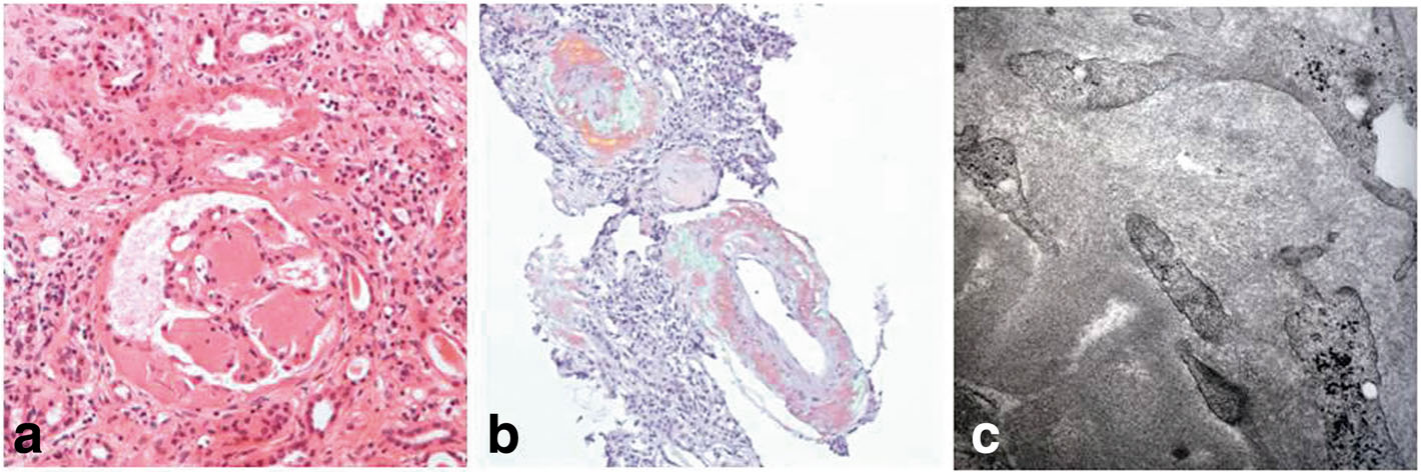
Renal biopsy findings. **a** Glomeruli and peripheral capillary wall contains segmental deposits of eosinophilic amorphous hyaline material (hematoxylin-eosin, × 100). **b** Congo red stain by birefringence under polarized light of arteriolar wall shows apple-green staining reaction (× 100). **c** Electron micrograph showing extensive infiltration by 10-nm fibrils

A slit lamp examination revealed superficial keratoconjunctival erosion in both eyes. A ^99m^Tc pertechnetate scan of her salivary gland showed normal function. A lip biopsy demonstrated amyloid deposition around her salivary ducts, and atrophy of her salivary duct (Fig. 2a, b). There was no mononuclear infiltration. A renal ultrasound scan showed increased echogenicity and the size of her right kidney and left kidney was 10.32 cm and 9.42 cm, respectively. A renal biopsy was performed. Light microscopy revealed a glomerular and peripheral capillary wall deposition of eosinophilic amorphous hyaline material (Fig. 3a). The Congo red stain by birefringence under polarized light showed apple-green staining reaction (Fig. 3b). Under electron microscopy, we observed non-branching, randomly distributed 10 nm-wide fibrils (Fig. 3c).

A bone marrow aspiration biopsy reveals hypercellular marrow with 5% plasma cells. She was diagnosed as having AL amyloidosis then started on chemotherapy with cyclophosphamide and steroids. A follow-up at 8 months showed that her renal function remained stable (creatinine 267.85 umol/L) with reduction in free kappa and the lambda light chain (0.119 g/L, 0.154 g/L). The nail dystrophy also improved without other treatment (Fig. 1b).

## Discussion

We report here a case of sicca syndrome and nail dystrophy with renal dysfunction in a 52-year-old Korean woman who was diagnosed as having systemic amyloidosis.

Mucocutaneous manifestations are occasionally found in amyloidosis. The most frequent skin and mucous membrane changes are purpura, petechiae, and ecchymoses. Moreover waxy papules, nodules or plaques, pigmentary changes, as well as scleroderma such as thickening of the skin and bullous lesions can be found [3]. These are sometimes the first clue to discover systemic involvement. In this presented case, our patient had severe dry mouth and nail dystrophy as her first symptoms.

Sicca syndrome consists of xerostomia and xerophthalmia caused by destruction of the salivary and lacrimal glands; it can be observed with a variety of diseases. The most common cause of sicca syndrome is Sjögren’s syndrome; Sjögren’s syndrome is an autoimmune disorder in which the salivary and lacrimal glands undergo progressive destruction by lymphoplasmacytic cells. At first, we presumed that in this present case our patient’s amyloidosis might be a secondary reaction of Sjögren’s syndrome but we could not find any lymphoplasmacytic infiltration in her lip and renal biopsy. Furthermore, the autoantibodies were all negative as well. Other less frequent causes of sicca syndrome are sarcoidosis, hemochromatosis, hyperlipoproteinemia, radiotherapy, acquired immunodeficiency syndrome, hepatitis C virus (HCV) infection, and graft versus host disease [4]. Many drugs such as anticholinergics, antidepressants, antipsychotic agents, analgesics, and antihistamines are also causes of sicca syndrome. We could exclude these causes by minutely examining our patient’s history, and by conducting a serology and autoimmune test, or biopsy.

A few cases of sicca syndrome as the initial symptom of primary amyloidosis have been reported in the literature. We comprehensively reviewed the English literature available in either abstract or full-text form that reported sicca syndrome in primary amyloidosis. The clinical characteristics of 14 patients with primary amyloidosis with sicca syndrome, including the present case, are summarized in Table 1 [3–15]. Among a total of 16 cases two cases were excluded from analysis because the languages were not English. In 14 cases, sicca syndromes represented the first manifestation of the disease. In seven of these cases, including our case, the patients presented additional skin or mucosal manifestations characteristic for amyloidosis. It is noteworthy that in seven of these patients, sicca syndromes were the sole cutaneous sign to the diagnosis of systemic amyloidosis. The duration of sicca syndrome from onset to diagnosis was 23.5 months (4–60 months). Therefore, the recognition of sicca syndrome as a manifestation of systemic amyloidosis is important because it can occasionally lead to an early diagnosis.

**Table 1:** Summary of reviewed data on primary amyloidosis with cutaneous manifestation

Case Number	Age/sex	Sicca syndrome as first manifestation/presence prior to diagnosis, months	Cutaneous involvement at time of diagnosis	Organ involvement	Underlying disease	Prognosis in the article/cause of death	Reported year (Reference)
1	52/F	Yes/4 months	Sicca syndrome and nail dystrophy	Kidney	No	Survived	Present case
2	71/M	Yes/12 months	Sicca syndrome	Kidney, heart, liver	No	Died (cholestatic hepatopathy)	1971 [5]
3	68/M	Yes/9 months	Sicca syndrome	Liver, kidney, heart, pancreas, GI tract, thyroid, prostate, adrenal, testes	No	Died (myocardial infarction)	1972 [6]
4	58/F	Yes/48 months	Sicca syndrome and bullous erythema multiform	Liver, heart, peripheral neuropathy	No	Died (unknown)	1983 [7]
5	63/M	Yes/(unknown)	Sicca syndrome	Kidney, heart	No	Died (heart failure)	1984 [8]
6	71/M	Yes/6 months	Sicca syndrome	Kidney	No	Survived	1988 [9]
7	66/F	Yes/24 months	Sicca syndrome and purpura, epidermal desquamation	Kidney, heart, lung, GI tract, liver, pancreas, adrenals, thyroid	No	Died (aspiration)	1991 [10]
8	74/F	Yes/(unknown)	Sicca syndrome	No	No	Survived	1992 [11]
9	49/M	Yes/(unknown)	Sicca syndrome, multiple ecchymoses	Kidney	Multiple myeloma	Died (GI bleeding, heart failure)	1993 [12]
10	52/F	Yes/ 24 months	Sicca syndrome	No	No	Survived	1994 [13]
11	79/M	Yes/48–60 months	Sicca syndrome, pinch purpura, easy bruising, nail dystrophy	Kidney	No	Survived	1996 [4]
12	76/M	Yes/6 months	Sicca syndrome	Kidney, heart, lung, peripheral neuropathy	No	Survived	1996 [14]
13	62/F	Yes/6 months	Sicca syndrome	Kidney	No	Survived	1998 [15]
14	66/M	Yes/60 months	Sicca syndrome, nail dystrophy, parchment-like hand changes, alopecia	Small fiber neuropathy	No	Survived	2014 [3]

*F* female, *GI* gastrointestinal, *M* male

Here, our patient also complained of nail dystrophy. Nail dystrophy is also a rare symptom of systemic amyloidosis and usually manifests as brittleness, longitudinal ridging, and splitting. In some cases, the biopsy shows amyloid deposits in the papillary dermis of the matrix and nail bed with nail involvement. A literature search revealed only 20 cases in which development of nail dystrophy was highlighted [3]. Unfortunately, we could not perform a nail biopsy; nail dystrophy in this present case improved after treatment of primary amyloidosis.

## Conclusions

The organs most commonly involved in primary amyloidosis are kidney, heart, and gastrointestinal tract. Less frequently, peripheral neuropathy, autonomic neuropathy, cholestatic hepatopathy, and infiltration of soft tissues including macroglossia can be found. The prognosis of primary amyloidosis is generally poor with death caused by renal or cardiac failure if the disease remains untreated. So the early diagnosis of systemic amyloidosis before disease progression is important. In summary, our observation reminds us that sicca syndrome and nail dystrophy may represent the leading manifestation of AL amyloidosis. Paying attention to these symptoms can lead to an early diagnosis of the disease.

## Abbreviations

RBC: Red blood cells
WBC: White blood cells
